# Mangrove derived *Streptomyces* sp. MUM265 as a potential source of antioxidant and anticolon-cancer agents

**DOI:** 10.1186/s12866-019-1409-7

**Published:** 2019-02-13

**Authors:** Loh Teng-Hern Tan, Kok-Gan Chan, Priyia Pusparajah, Wai-Fong Yin, Tahir Mehmood Khan, Learn-Han Lee, Bey-Hing Goh

**Affiliations:** 1 Institute of Biomedical and Pharmaceutical Sciences, Guangdong University of Technology, Guangzhou, 510006 People’s Republic of China; 2 Biofunctional Molecule Exploratory (BMEX) Research Group, School of Pharmacy, Monash University Malaysia, 47500 Bandar Sunway, Selangor Darul Ehsan Malaysia; 3 Novel Bacteria and Drug Discovery (NBDD) Research Group, Microbiome and Bioresource Research Strength, Jeffrey Cheah School of Medicine and Health Sciences, Monash University Malaysia, 47500 Bandar Sunway, Selangor Darul Ehsan Malaysia; 4 International Genome Centre, Jiangsu University, Zhenjiang, China; 5 Division of Genetics and Molecular Biology, Faculty of Science, Institute of Biological Sciences, University of Malaya, Kuala Lumpur, Malaysia; 6 Medical Health and Translational Research Group, Jeffrey Cheah School of Medicine and Health Sciences, Monash University Malaysia, 47500 Bandar Sunway, Selangor Darul Ehsan Malaysia; 7 Institute of Pharmaceutical Science, University of Veterinary and Animal Science, Lahore, Pakistan; 8 Center of Health Outcomes Research and Therapeutic Safety (Cohorts), School of Pharmaceutical Sciences, University of Phayao, Phayao, Thailand

**Keywords:** *Streptomyces*, Mangroves, Antioxidant, Cytotoxic, Colon cancer

## Abstract

**Background:**

Colon cancer is the third most commonly diagnosed cancer worldwide, with a commensurately high mortality rate. The search for novel antioxidants and specific anticancer agents which may inhibit, delay or reverse the development of colon cancer is thus an area of great interest; *Streptomyces* bacteria have been demonstrated to be a source of such agents.

**Results:**

The extract from *Streptomyces* sp. MUM265— a strain which was isolated and identified from Kuala Selangor mangrove forest, Selangor, Malaysia— was analyzed and found to exhibit antioxidant properties as demonstrated via metal-chelating ability as well as superoxide anion, DPPH and ABTS radical scavenging activities. This study also showed that MUM265 extract demonstrated cytotoxicity against colon cancer cells as evidenced by the reduced cell viability of Caco-2 cell line. Treatment with MUM265 extract induced depolarization of mitochondrial membrane potential and accumulation of subG_1_ cells in cell cycle analysis, suggesting that MUM265 exerted apoptosis-inducing effects on Caco-2 cells.

**Conclusion:**

These findings indicate that mangrove derived *Streptomyces* sp. MUM265 represents a valuable bioresource of bioactive compounds for the future development of chemopreventive agents, with particular promise suggested for treatment of colon cancer.

**Electronic supplementary material:**

The online version of this article (10.1186/s12866-019-1409-7) contains supplementary material, which is available to authorized users.

## Background

Colon cancer is a global concern, ranking 3rd out of 27 common cancers across 184 countries [1]; while it once viewed as a disease affecting developed countries, recent data suggests that the incidence of colon cancer is increasing among the Asia-Pacific population. A variety of possible reasons have been postulated to explain this observation including increasing life expectancy, adoption of Western lifestyle [2] as well as lifestyle changes occurring in parallel with economic development including diet-related factors [3], physical inactivity, obesity, smoking, and alcohol consumption [4, 5]. Worldwide mortality attributable to colorectal cancer is approximately half that of the incidence; colorectal cancer is the fourth most common cause cancer related deaths worldwide [4]. Given these statistics, new drugs that have the potential to delay or prevent malignant growths in the colon therefore potentially reducing the incidence and therefore mortality rates would be of tremendous value.

The new molecules we seek may come from an unlikely source: bacteria from mangrove swamps, which are the woody plant areas in the intertidal coasts in tropical and subtropical coastal regions. These areas are home to a diverse array of unique microorganisms; and represents an excellent target in the hunt for novel metabolites as the mangrove ecosystem places constant environmental stressors on its inhabitants with its constant changes of salinity and tidal gradients [6, 7] which then drives metabolic pathway adaptations for survival among the mangrove-associated microorganisms. These metabolic adaptations are likely to result in the synthesis of medically valuable, bioactive metabolites [8–10]. Exploration of the mangrove is all the more exciting as recently many new species have been discovered suggesting the huge untapped potential of this environment [11, 12]. Work on this has so far resulted in the discovery of several novel *Streptomyces* species from mangrove environments on a global scale including *S. shenzhenensis* [13], *S. pluripotens* [14], *S. ferrugineus* [15], *S. mangrovisoli* [16], *S. gilvigriseus* [17], *S. malaysiense* [18], *S. humi* [19], *S. antioxidans* [20] and *S. colonosanans* [21].

The discovery of novel *Streptomyces* species is particularly exciting as *Streptomyces* are a prolific source of various natural products with diverse biological activities [22, 23]. Notably, *Streptomyces* is a producer of many clinically important drugs, including the anticancer agents doxorubicin [24] and bleomycin [25], as well as the antifungal agent nystatin [26]. It stands to reason that the *Streptomyces* species derived from previously unexplored environments such as the mangrove ecosystem are highly likely to be producers of valuable secondary metabolites with interesting bioactivities [9, 27–30]. Conventionally, plants have been heralded as a rich source of antioxidants which have potential to treat many diseases including cancer [31–33] and much work in drug discovery has focused on them; but recently, work with microorganisms has demonstrated that they may also represent a rich source of natural antioxidants. Furthermore, recent research has revealed that mangrove *Streptomyces* produce metabolites with antioxidative activity [16, 29, 34]. Previous studies have reported that *Streptomyces* sp. produce a number of bioactive compounds with anticancer and antitumor properties, particularly against colon cancer - piperazimycins [35], pladienolides [36], and calcimycin [37] are some of the bioactive compounds isolated from *Streptomyces* sp. that exhibit cytotoxicity toward human colon cancer cells. Research findings have also suggested that *Streptomyces* is potentially a good source of chemopreventive agents [18, 38, 39]. The chemopreventive properties of phenazine compounds isolated from a marine-derived *Streptomyces* sp. have been extensively studied and were demonstrated to have chemopreventive potential, as evidenced by inhibition of quinone reductase 2 (phase II enzymes), induction of quinone reductase 1 (phase I enzymes), inhibition of cyclooxygenase and induction of apoptosis through subG1 phase cell cycle arrest [38, 39].

The aim of this study is to explore the biological activity of *Streptomyces* sp. MUM265, which our team isolated from soil samples from the Kuala Selangor mangrove forest on the west coast of Peninsula Malaysia. This work represents part of our bioprospecting effort to explore the potential biological activities of new strains of *Streptomyces*, focusing on their antioxidant and cytotoxic properties. The results we obtained indicate that MUM265 extract possesses significant antioxidant properties and is cytotoxic against colon cancer cells. GC-MS chemical profiling identified several bioactive chemical compounds present in the extract of MUM265. A good correlation was observed between the chemical constituents identified from MUM265 extract and its antioxidant and cytotoxic properties. Thus, the outcomes derived from this research continue to build on the foundation supporting the continued bioprospecting of mangrove derived *Streptomyces* as sources of potential chemopreventive agents in greater depth*,* with a particular focus on the development of chemopreventive drugs for colon cancer.

## Results

### Strain identification using 16S rRNA-based phylogenetic analysis

**Fig. 1:**
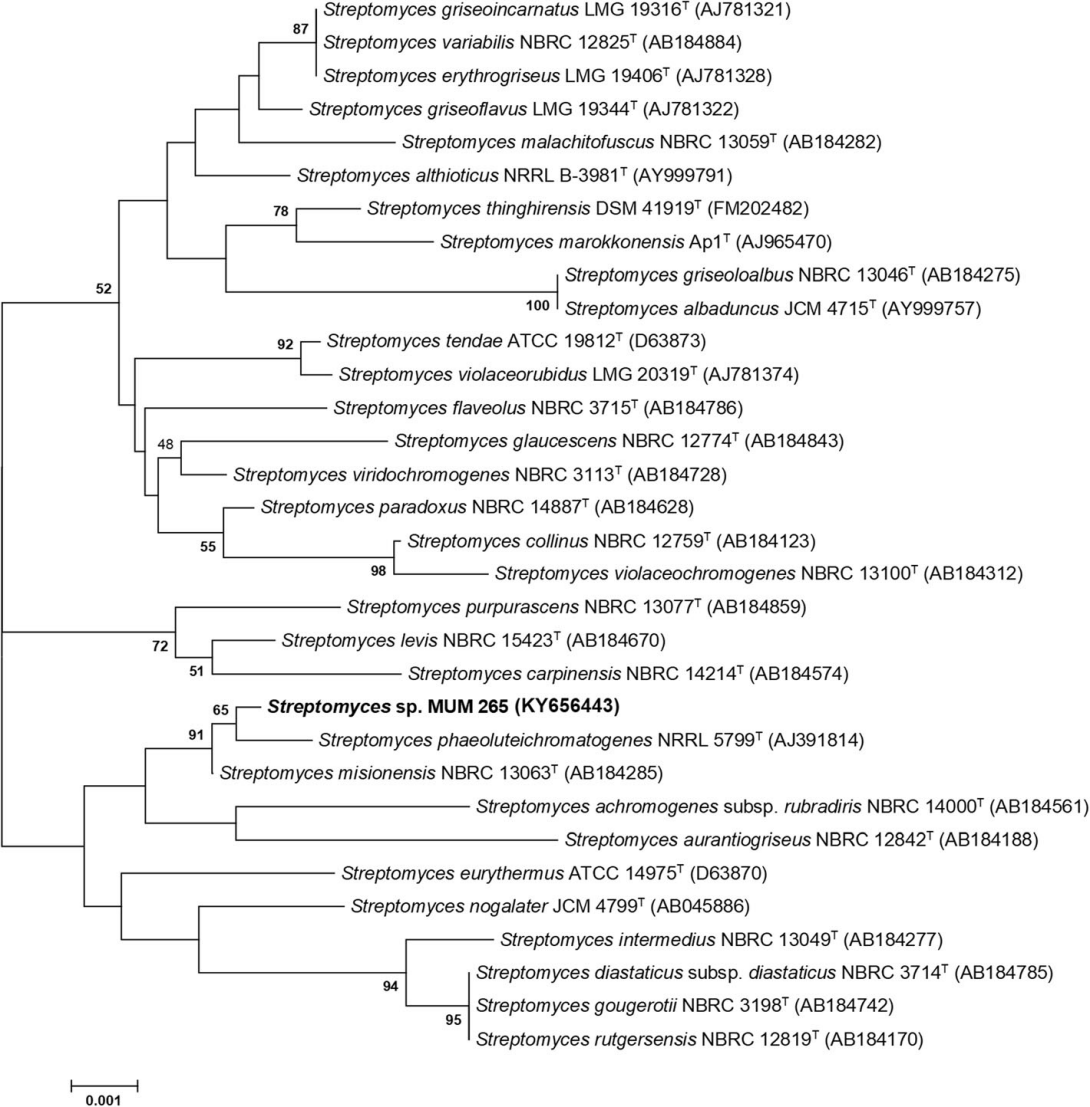
Neighbour-joining phylogenetic tree based on 16S rRNA sequences of strain MUM265 and other related taxa. Bootstrap values (> 50%) based on 1000 re-sampled datasets are shown at branch nodes. Bar, 0.001 substitutions per site

The sequencing result revealed a 1340 bp 16S rRNA gene sequence of strain MUM265 which has been submitted and deposited in GenBank with accession number (KY656444). Figure 1 shows a 16S rRNA gene phylogenetic tree of strain MUM265 based on neighbor-joining method which demonstrated a single clade formed between strain MUM265 and another two type strains, including the *Streptomyces misionensis* NBRC 13063^T^ and *Streptomyces phaeoluteichromatogenes* NRRL 5799^T^. This clade is also supported by the high bootstrap value of 91%, showing a high confidence level of the association. Strain MUM265 was found to be closely related with *Streptomyces misionensis* NBRC13603^T^ (99.8%) and *Streptomyces phaeoluteichromatogenes* NRRL 5799^T^ (99.8%) with high 16S rRNA gene sequence similarity.

### Phenotypic characterization of strain MUM265

**Fig. 2:**
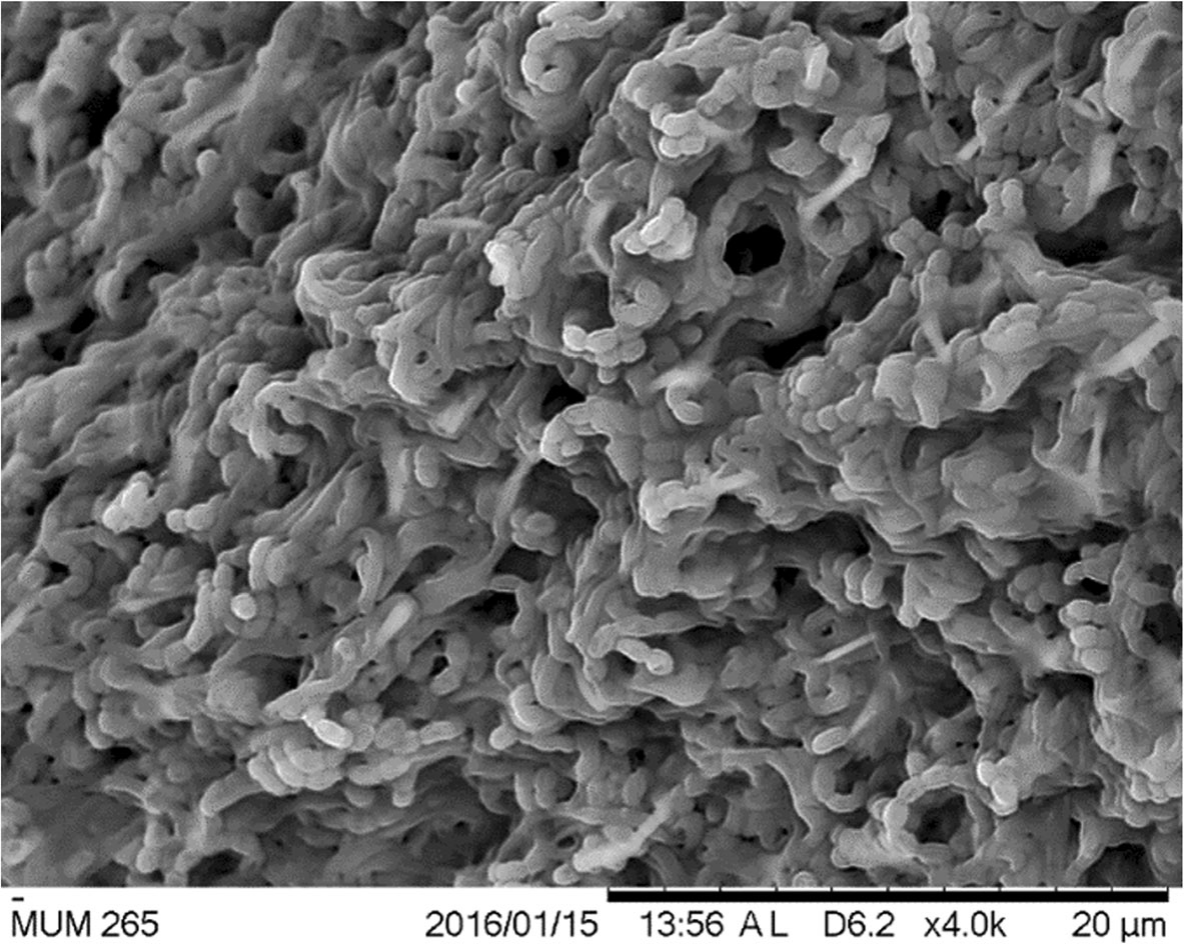
The scanning electron micrographs of *Streptomyces* sp. MUM265. It appears as filaments and branch to form a network of filaments called mycelium. Spiral shape spore chains are also clearly visible in SEM micrographs

*Streptomyces* sp. MUM265 is Gram-positive and aerobic. On ISP2 agar, it forms brilliant yellow aerial and light-yellow substrate mycelium. The color of *Streptomyces* sp. MUM265 colony varies on different media. The strain grows well on all the agar tested after 2 weeks at 28 °C, except on ISP 4 agar (no growth). When subjected to scanning electron microscopy, strain MUM265 was shown to produce spiral spore chains (Fig. 2). These morphological characteristics also strongly indicated that strain MUM265 belonged to the genus *Streptomyces* [40]. Strain MUM265 was also shown to be negative for haemolytic activity as no clear zone was observed around the colonies formed on the blood agar. The biochemical and growth characteristics of strain MUM265 cultured under different growth conditions are tabulated in Table 1. An interesting additional finding of ours was that strain MUM265 hydrolyzes starch and cellulose, indicating that strain MUM265 possesses the potential to be an industrially important strain that produces enzymes which are valuable for the pharmaceutical and food industries [41].

**Table 1:** The phenotypic and physiological characteristics of *Streptomyces* sp. MUM265

Characteristics	Strain MUM265
Tolerance to NaCl	Up to 6% (w/v) (optimum at 2% (*w*/*v*))
Temperature optimum	Up to 40 °C (36 °C)
pH optimum	6–7
Melanin production	–
Haemolytic activity	γ (gamma)/negative
Hydrolysis of:	
Carbomethylcellulose	+
Casein	–
Catalase	+
Chitin	–
Starch	+
Tributyrin	–
Xylan	–

Physiological factors and carbon sources are important determinants influencing secondary metabolite production in *Streptomyces* species [42, 43]. Small manipulations of nutritional or environmental factors during cultivation may significantly impact the quantity of a particular compound that is produced or even the general metabolic profile of the *Streptomyces* species. The physiological tests conducted revealed that strain MUM265 exhibits moderate salt and temperature tolerance (up to 6% *w*/*v* and 40 °C respectively). This is in agreement with other studies on mangrove derived *Streptomyces* strains that also demonstrated tolerance to moderate to high NaCl concentrations ranging from 6 to 10% (w/v) [44, 45].

The biosynthesis of secondary metabolites in *Streptomyces* sp. is significantly dependent on the availability of carbon and nitrogen sources; therefore varying the composition of substrates provided can result in the production of a varied quantity of compounds and also the production of distinct groups of molecules [42, 46]. Thus, the carbon and nitrogen source utilization of strain MUM265 was assessed by employing the Biolog GEN III MicroPlate system to obtain an overview of its metabolic profile. Strain MUM265 was shown to have the capability to use a great variety of different substrates. The results are interpreted and summarized in Table 2. The system also showed that growth of strain MUM265 is not inhibited by azetreonam, guanidine HCl, rifamycin RV, tetrazolium blue, tetrazolium violet, potassium tellurite, sodium butyrate, sodium bromate and 1% sodium lactate. Moreover, the antibiotic susceptibility test showed that strain MUM265 is resistant to penicillin G, ampicillin and nalidixic acid but could not be inhibited by vancomycin, erythromycin, chloramphenicol, ampicillin-sulbactam, cefotaxime, gentamicin and tetracycline (see Additional file 1).

**Table 2:** The utilization of carbon and nitrogen sources by *Streptomyces* sp. MUM265

Utilizable carbon and nitrogen sources		Non-utilizable carbon and nitrogen sources	
Acetic acid	α-D-glucose	3-methylglucose	Acetoacetic acid
α-hydroxy-butyric acid	α-keto-glutaric acid	α-D-lactone	β-methyl-D-glucoside
β-hydroxy-D,L-butyric acid	Bromo-succinic acid	D-aspartic acid	D-glucose-6-phosphate
Citric acid	D-arabitol	D-lactic acid methyl ester	D-malic acid
D-cellobiose	Dextrin	D-melibiose	D-raffinose
D-fructose	D-fructose-6-phosphate	D-saccharic acid	D-salicin
D-fucose	D-galactose	D-serine	D-sorbitol
D-galacturonic acid	D-gluconic acid	D-turanose	L-rhamnose
D-glucuronic acid	D-maltose	Mucic acid	N-acetyl-D-galactosamine
D-mannitol	D-mannose	N-acetyl-neuraminic acid	N-acetyl-β-D-mannosamine
D-trehalose	Formic acid	ρ-hydroxy-phenylacetic acid	Quinic acid
γ-amino-butyric acid	Gelatin	Stachyose	Sucrose
Gentiobiose	Glucuronamide		
Glycerol	Glycyl-L-proline		
Inosine	L-alanine		
L-arginine	L-aspartic acid		
L-fucose	L-galactonic acid lactone		
L-glutamic acid	L-histidine		
L-lactic acid	L-malic acid		
L-pyroglutamic acid	L-serine		
Methyl pyruvate	myo-inositol		
N-acetyl-D-glucosamine	Pectin		
Propionic acid	Tween 40		

### Antioxidant activities of MUM265 extract

To evaluate the antioxidant potential of *Streptomyces* sp. MUM265 extract, four different antioxidant assays were conducted. The results of its antioxidant activity as measured by the different assays are presented in Table 3.

**Table 3:** The antioxidant activities demonstrated by *Streptomyces* sp. MUM265 extract and positive controls in different antioxidant assays

Concentration of extract / positive controls (μg/mL)	Antioxidant activities			
	DPPH radical scavenging activity (%)	ABTS radical scavenging activity (%)	Superoxide dismutase-like activity (%)	Metal-chelating activity (%)
MUM265 extract				
62.5	ND^a^	5.31 ± 2.21	9.73 ± 5.72^*^	12.57 ± 2.10^*^
125	ND^a^	9.40 ± 3.17^*^	16.84 ± 4.48^*^	18.55 ± 1.13^*^
250	1.22 ± 1.47	16.42 ± 4.20^*^	17.67 ± 2.52^*^	22.53 ± 0.70^*^
500	9.43 ± 1.58^*^	24.29 ± 4.01^*^	24.20 ± 1.98^*^	24.32 ± 3.56^*^
1000	24.70 ± 3.12^*^	46.56 ± 4.95^*^	28.59 ± 1.79^*^	28.86 ± 6.27^*^
2000	33.69 ± 3.36^*^	59.13 ± 6.14^*^	37.25 ± 0.21^*^	34.06 ± 1.28^*^
4000	42.33 ± 3.98^*^	88.50 ± 0.37^*^	55.99 ± 1.03^*^	46.02 ± 0.86^*^
Gallic acid				
6.25	32.13 ± 5.48^*^	–	–	–
12.5	–	85.77 ± 4.50^*^	–	–
Catechin				
0.3	–	–	52.27 ± 6.27^*^	–
EDTA				
62.5	–	–	–	53.32 ± 4.57^*^

^*^statistically significance (*P* < 0.05) when compared to control (without extract)

^a^*ND* not detected

Our results showed that *Streptomyces* sp. MUM265 extract exhibited significant DPPH scavenging activity over a range of extract concentrations from 0.5 to 4 mg/mL (*p* < 0.05) with a 9.43 ± 1.22% to 42.33 ± 3.99% reduction of DPPH radicals. The radical scavenging activity of *Streptomyces* sp. MUM265 extract was also evaluated using another stable radical cation, ABTS^●+^. This assay showed that *Streptomyces* sp. MUM265 extract scavenged ABTS radical with capacity measuring from 9.40 ± 3.17% to 88.50 ± 0.37% at extract concentrations ranging from 0.125 mg/mL to 4 mg/mL. The ability of *Streptomyces* sp. MUM265 extract to scavenge superoxide anion radical (O_2_^●-^) was assessed by detecting the reduction of O_2_^●-^ level with WST-1 for the determination of SOD-like activity [47]. The assay revealed that *Streptomyces* sp. MUM265 extract demonstrated significant superoxide anion scavenging activity ranging from 9.73 ± 5.72% to 55.99 ± 1.03% over a range of extract concentrations from 0.0625 mg/mL to 4 mg/mL (*p* < 0.05). The metal-chelating activity of *Streptomyces* sp. MUM265 extract was evaluated by measuring the reduction of ferrozine-Fe^2+^ complex formation. This assay showed that *Streptomyces* sp. MUM265 extract possesses significant metal chelating activity in terms of inhibiting the formation of ferrozine-Fe^2+^ complex, measuring from 12.57 ± 2.10% to 46.02 ± 0.86% at extract concentrations tested from 0.0625 mg/mL to 4 mg/mL.

Studies have shown that phenolic compounds and flavonoids -both effective antioxidants - are naturally present in microorganisms [48–50]. On this basis, we chose to perform assays to determine the total phenolic and flavonoid content of the extract. We also performed a correlation analysis to evaluate the relationship between the antioxidant capacity of MUM265 extract and its total phenolic content (TPC). TPC of *Streptomyces* sp. MUM265 extract was measured using Folin-Ciocalteu’s reagent and the result was suggestive of the presence of phenolic compounds as TPC increased with increasing extract concentration. Meanwhile, a negative result was shown by the flavonoid content assay, suggesting either that this assay was not sensitive enough to detect flavonoids or that the flavonoid concentration in the *Streptomyces* sp. MUM265 extract was too low to be detected. Table 4 shows the Pearson’s correlation coefficients between the variables. The strongest positive correlation was observed between the TPC and SOD-like activity of *Streptomyces* sp. MUM265 extract (*r* = 0.991, *p* < 0.05). The strong correlation between the four assays measuring antioxidant capacity and the total phenolic content indicates that phenolic compounds are likely to be a major contributor to the antioxidant properties of *Streptomyces* sp. MUM265 extract. Phenolic compounds are widely recognized as potent chemopreventive agents, with their mechanism of action believed to hinge on their antioxidant action and modulation of intracellular signaling pathways associated with the initiation and promotion of carcinogenesis [51].

**Table 4:** Pearson’s correlation coefficients between TPC and antioxidant activities of *Streptomyces* sp. MUM265 extract

Antioxidant activities	Phenolic content
DPPH radical scavenging activity	*r* = 0.941^a^
ABTS radical scavenging activity	*r* = 0.982^a^
SOD-like activity	*r* = 0.991^a^
Metal-chelating activity	*r* = 0.980^a^

^a^Correlation is significant at the 0.05 level

### Effect of MUM265 extract on cell viability of cancer and normal cells

The cytotoxicity of *Streptomyces* sp. MUM265 extract was evaluated against various colon cancer cells. Cell viability post exposure to variable concentrations of extract was assessed via an MTT assay. CCD-18Co cells were included to evaluate the toxicity of the extract against normal cells, which served as a measure of the selectivity of the extract against cancer cells vs normal cells, a key and highly desirable quality in any chemotherapeutic agent. CCD-18Co is a human fibroblast cell line isolated from normal colon tissue and has been widely used as normal control in cancer studies [52–54].

**Fig. 3:**
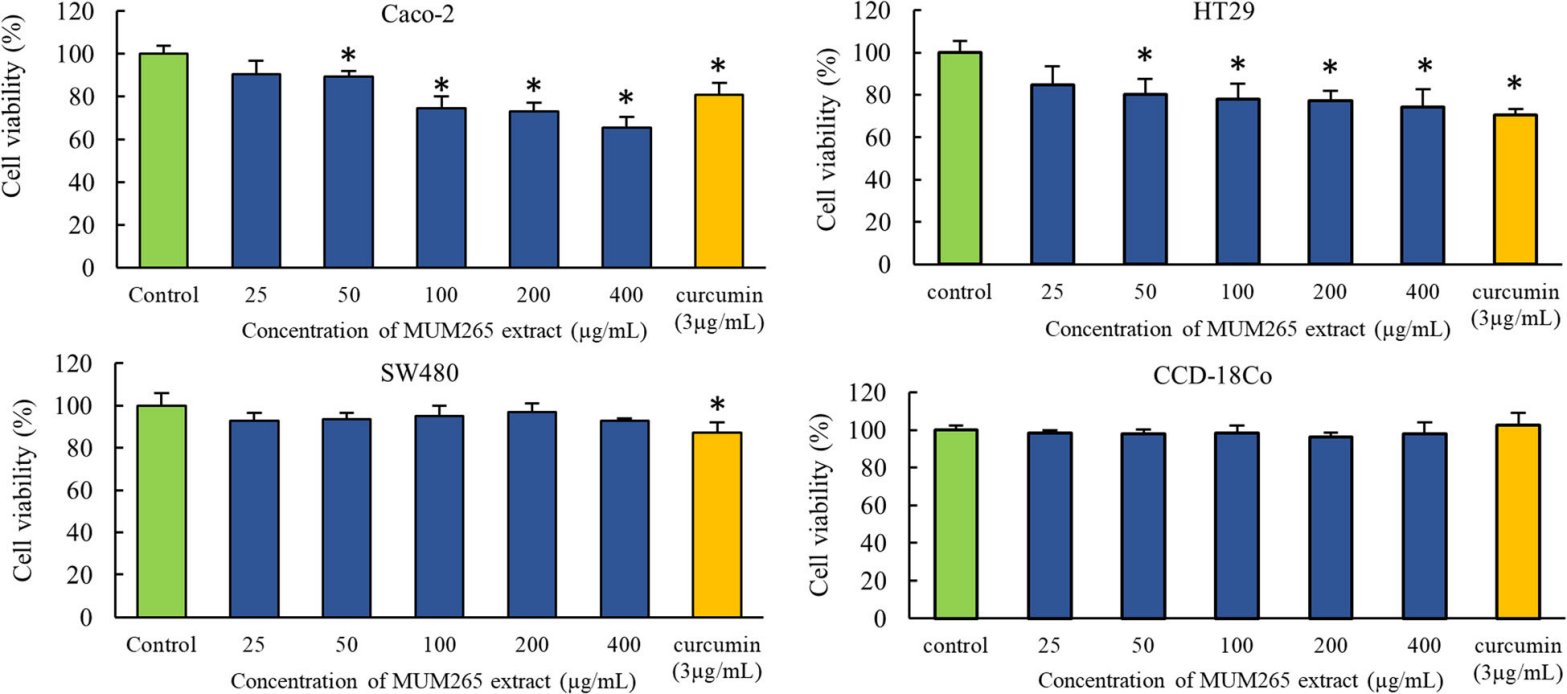
Cytotoxicity of *Streptomyces* sp. MUM265 extract against human colon cancer cell lines. The cytotoxicity of *Streptomyces* sp. MUM265 extract against the colon cancer cell lines and non-cancerous CCD-18Co cell measured using MTT assay. Each bar represents the mean of the cell viability of the cell lines after exposure to extract at respective concentrations tested for 72 h (*n* = 5). The vertical lines associated with the bars represent the SD of the mean. Symbol (*) indicates P < 0.05 significant difference compared to control (0.5% DMSO). Curcumin (3 μg/mL) was used as the positive control

The cytotoxicity of MUM265 extract is depicted in Fig. 3, demonstrating the cell viability of each colon cell line after exposure to MUM265 extract across a range of final concentrations for 72 h. The cytotoxicity test revealed that *Streptomyces* sp. MUM265 extract in concentrations ranging from 50 to 400 μg/mL resulted in significant reduction of the cell viability of both HT29 and Caco-2 cells. Caco-2 was the most sensitive toward the *Streptomyces* sp. MUM265 extract among the tested cell lines, as determined by the highest reduction in cell viability of Caco-2 (*p* < 0.05) of 34.57 ± 4.99% noted at 400 μg/mL extract concentration. Conversely, SW480 was not sensitive toward *Streptomyces* sp. MUM265 extract. There was no significant reduction in cell viability of normal colon cells, CCD-18Co treated with *Streptomyces* sp. MUM265 extract at concentrations tested from 25 to 400 μg/mL. This also implies that *Streptomyces* sp. MUM265 extract within the concentrations tested is non-toxic toward non-cancerous cells but exerts cytotoxicity against both Caco-2 and HT29 cancer cells. This result also suggests that among various colon cancer cell lines, *Streptomyces* sp. MUM265 extract exhibits a preferential cytotoxicity against both HT29 and Caco-2 (*p* < 0.05). However, ED_50_ (dose that is required to induce 50% reduction in cell viability) was not achieved for MUM265 extract even at the highest concentration tested (400 μg/mL) in the study. Higher concentration of MUM265 extract was not used for further investigation due to the nature of the extract as its solubility limit posed a challenge for the in vitro experimental set-up.

**Fig. 4:**
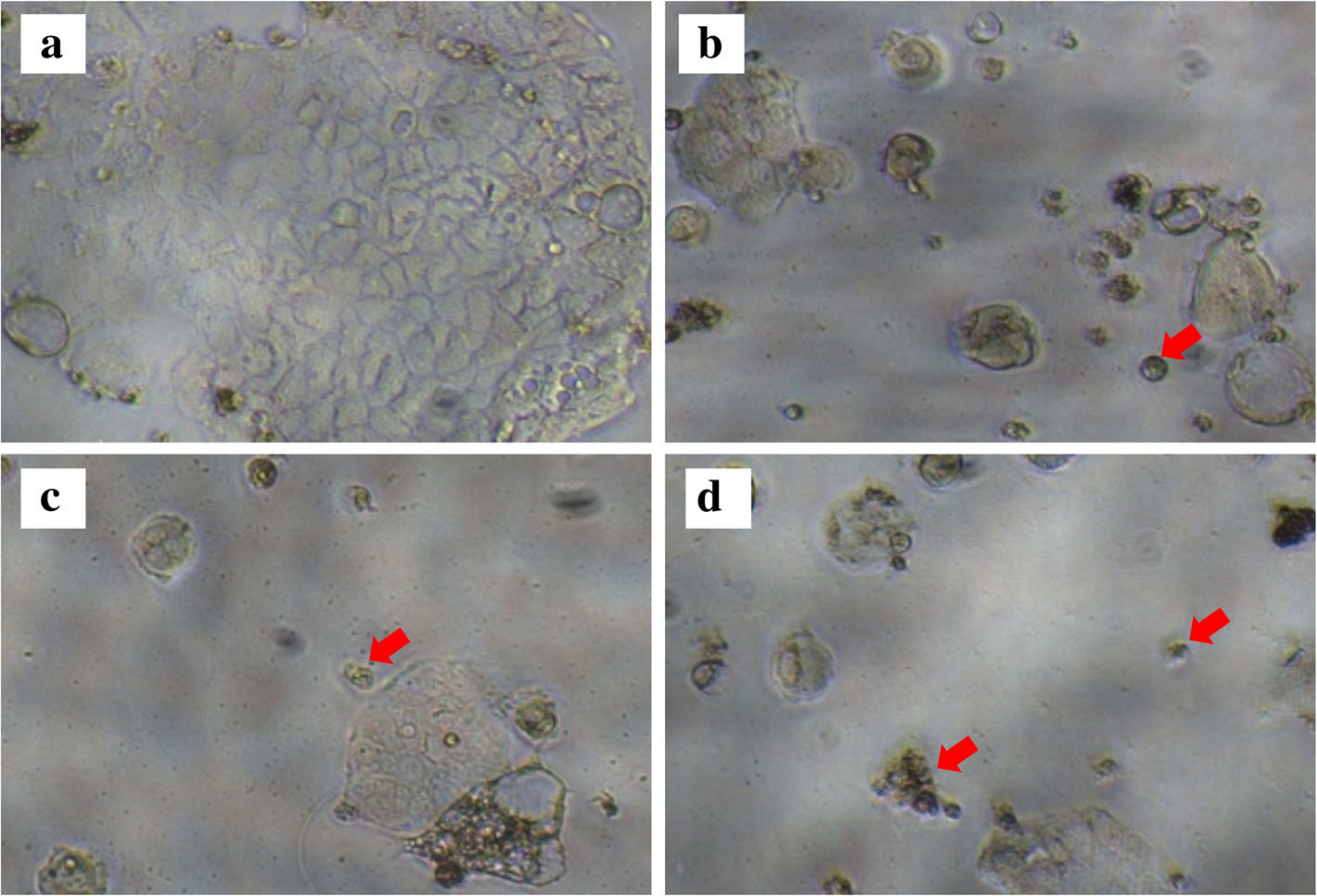
Morphology of Caco-2 cells after treatment with and without *Streptomyces* sp. MUM265 extract. Comparison of the morphological features of Caco-2 after the 72 h incubation without *Streptomyces* sp. MUM265 extract (0.5% DMSO) (**a**) and with the extract (**b**, **c** and **d**) observed under an inverted microscope with objective lens × 40. Arrow indicates the abnormal morphological features resulted from the cytotoxic effect of MUM265 extract (**b**, **c** and **d**)

In addition to measuring cell viability via MTT assay, morphological evaluation of the cancer cells was performed to investigate the effect of the extract on the appearance of the cells. As depicted in Fig. 4, treatment with *Streptomyces* sp. MUM265 extract visibly altered the cell morphology of the viable Caco-2 cells. The untreated Caco-2 cells appeared polygonal shaped with some vesicle-like structures (a), however majority of the treated cells showed abnormal morphological features. Treatment with MUM 265 extract caused rounding and detachment of Caco-2 cells. Additionally, cell shrinkage with reduced cytoplasm mass was also observed (indicated by arrows) upon treatment as depicted in Fig. 4d.

### Effect of MUM265 extract on DNA content of Caco-2 cells

**Fig. 5:**
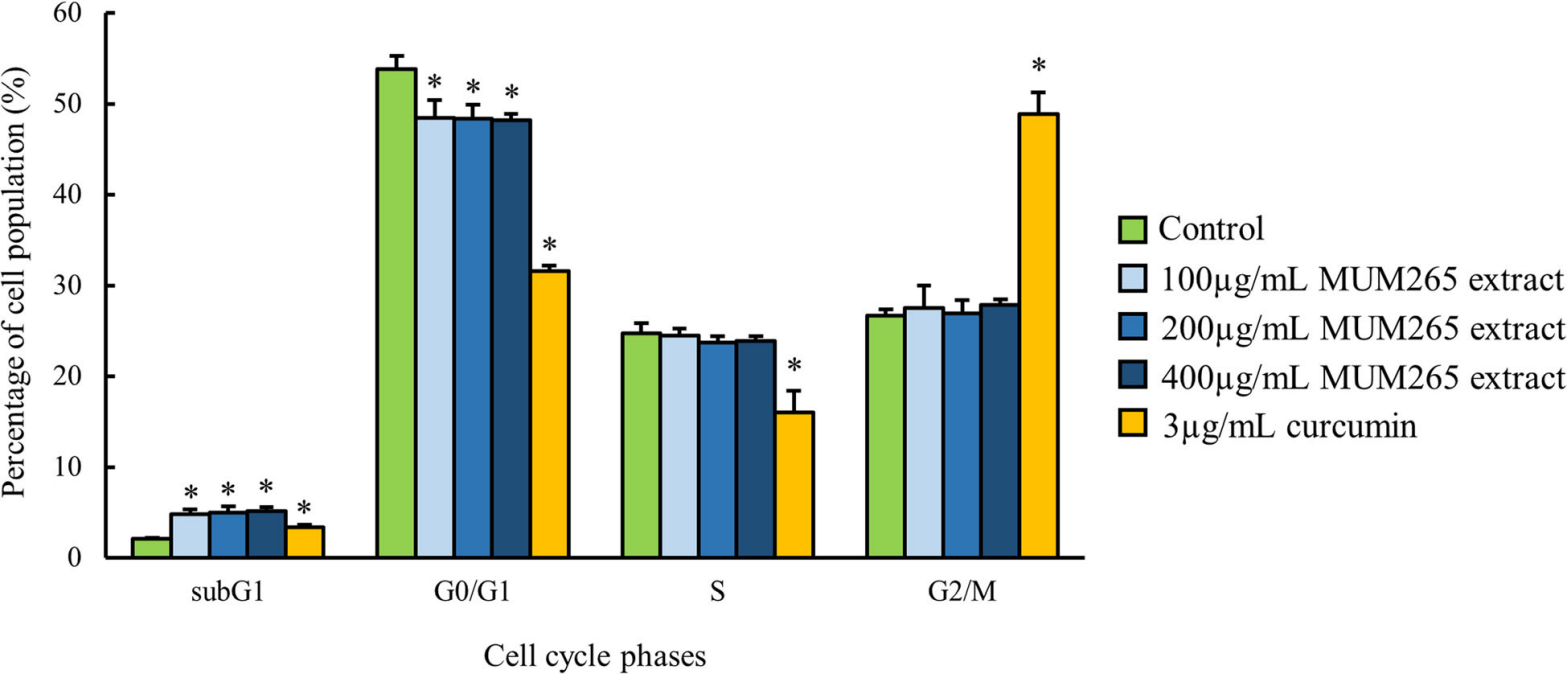
Effect of MUM265 extract on DNA distribution pattern of Caco-2 cells. PI fluorescent intensity was analyzed using flow cytometry. DNA distribution in different phases of cells followed by 24 h exposure to extract concentrations ranging from 100 μg/mL to 400 μg/mL. The data represents mean ± S.D. of quadruplicates (*n* = 4). Symbol (*) indicates *p* < 0.05 significant difference compared to control (0.5% DMSO). Curcumin (3 μg/mL) was used as positive control

Intranucleosomal DNA fragmentation is a major hallmark of apoptosis [55, 56]. Thus, we assessed the effect of treatment with MUM265 extract on DNA content of PI-stained Caco-2 cells using flow cytometry. PI is a fluorescent dye that binds stoichiometrically to nucleic acids [57]; and as fluorescence emission is proportional to DNA content of the cells, it allows the analysis of a cell population’s replication state. Hence, cells undergoing apoptosis can be determined from the DNA content histograms as those with lower fluorescent signal due to the low molecular weight of DNA fragments present in these cells compared to the G1 cells [56]. Upon exposure to MUM265 extract for 24 h, the percentage of Caco-2 cells in G0/G1 phases decreased with a concomitant increase of cells in the sub-G_1_ phase (Fig. 5).

### Effect of MUM265 extract on mitochondrial membrane potential

**Fig. 6:**
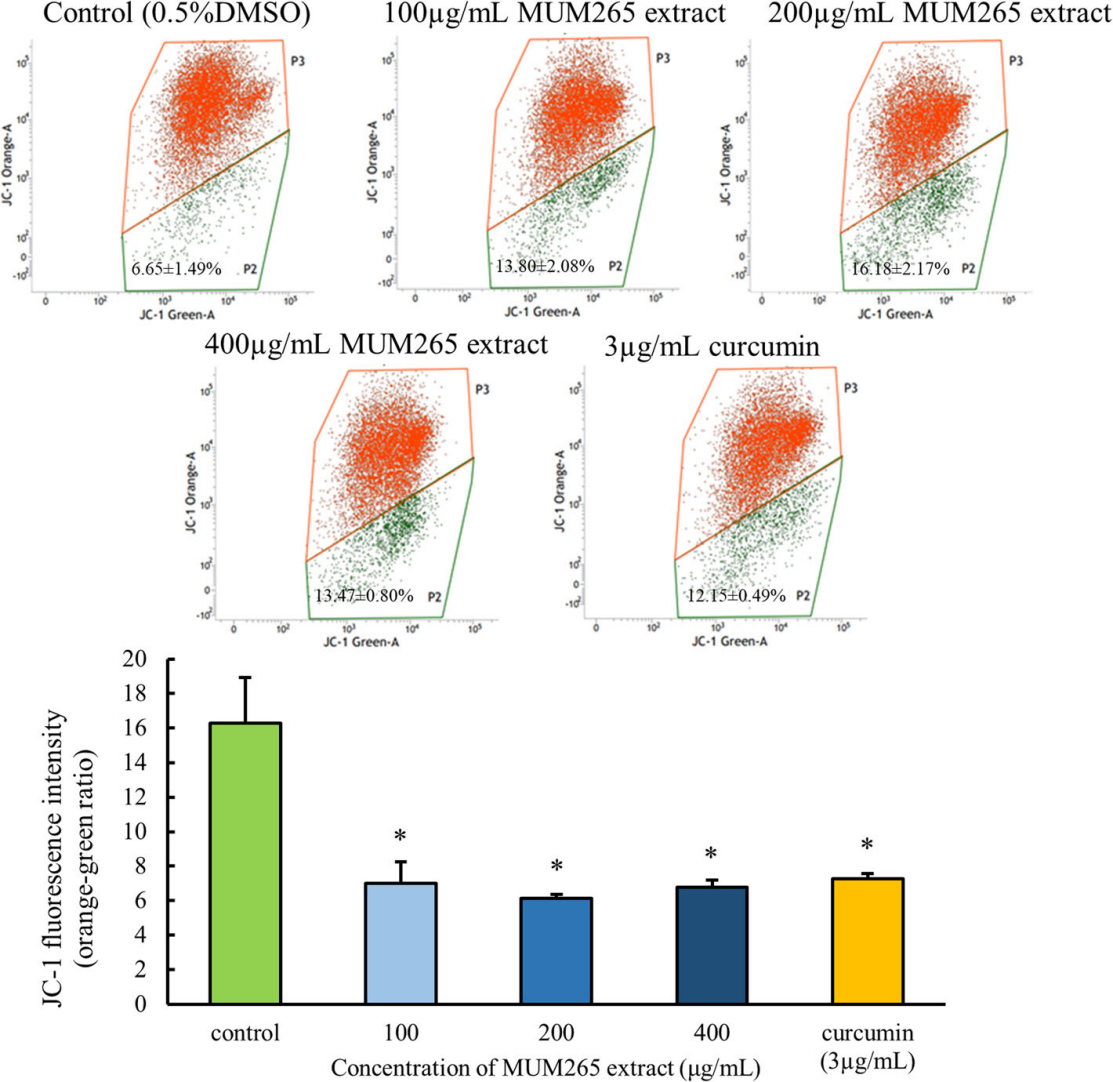
Flow cytometric analysis of MUM265 extract on MMP in Caco-2 cells. a) Representative dot plots of JC-1 aggregates (orange fluorescence) versus JC-1 monomers (green fluorescence). b) The bar chart shows that MUM265 extract has induced the reduction of orange/green fluorescence corresponding to the loss of MMP. The data expressed as mean ± S.D. of quadruplicates (*n* = 4). Symbol (*) indicates *p* < 0.05 significant difference compared to control (0.5% DMSO). Curcumin (3 μg/mL) was used as positive control

JC-1 dye exhibits variable characteristics when bound to the membrane of apoptotic cells versus non-apoptotic cells. In non-apoptotic cells, JC-1 dye appears in aggregate form emitting orange fluorescence at 590 nm when bound on polarized mitochondrial membrane with high membrane potential; but exists in monomeric form that emits green fluorescence at 527 nm in cells with disrupted mitochondrial potential [58]. The result of flow cytometry showed that treatment of Caco-2 cells with MUM265 extract resulted in the increase of green-fluorescence-positive cells, as shown in the P2 region (Fig. 6). As compared to untreated Caco-2 cells (control) (0.5% DMSO), a significant decrease (*p* < 0.05) in the ratio of orange-green fluorescence intensity was observed in all concentrations of MUM265 extract tested, indicating depolarization of mitochondrial membrane potential (Fig. 6). Therefore, this result suggested that the MUM265 extract induced cell apoptosis by causing the collapse of mitochondrial membrane potential (MMP) in Caco-2 cells. Taken together, the result of MTT assay is consistent with the observed reduced MMP and accumulated sub-G_1_ cell population in Caco-2 cells exposed to MUM265 extract, suggesting that the reduced cell viability of Caco-2 cells upon MUM265 extract treatment is the result of an apoptosis-inducing effect.

### GC-MS profiling of MUM265 extract

**Fig. 7:**
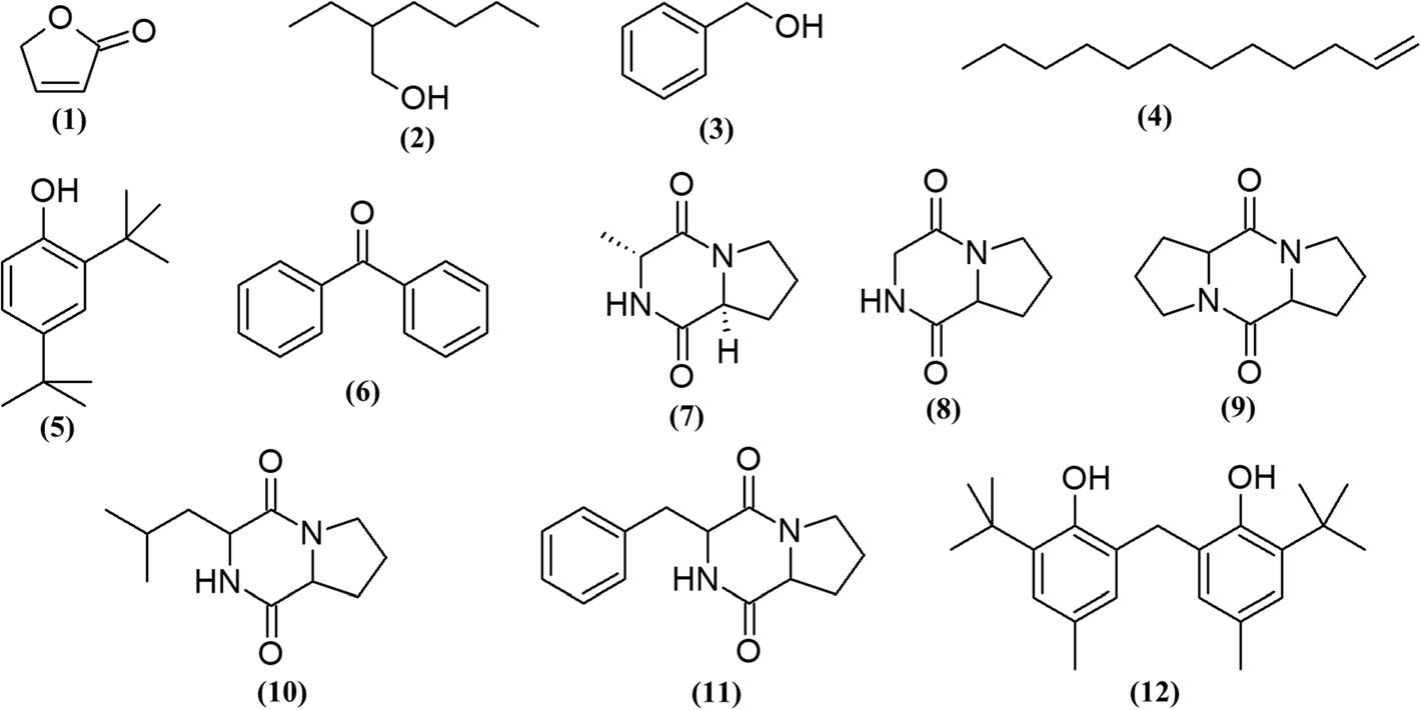
Chemical structures of constituents detected in MUM265 extract

In order to identify some of the chemical constituents potentially responsible for the observed antioxidant and cytotoxic effects, the *Streptomyces* sp. MUM265 extract was subjected to GC-MS analysis. Our analysis detected a variety of compounds including hydrocarbons, alcohols, phenolics and cyclic dipeptides present in the complex mixturethat is *Streptomyces* sp. MUM265 extract. Table 5 lists the detailed information of the identified chemical constituents. Figure 7 depicts the chemical structures of the constituents. In addition, the mass spectrum of the constituents **(1–12)** identified from the GC-MS analysis are provided (see Additional file 1).

**Table 5:** Chemical constituents identified in of *Streptomyces* sp. MUM265 extract

No.	Constituents {synonyms}	Retention time (min)	Molecular formula	Molecular Weight (MW)	Similarity (%)
1	2(5H)-furanone	13.844	C_4_H_4_O_2_	84	90
2	2-ethyl hexan-1-ol	21.466	C_8_H_18_O	130	83
3	Benzyl alcohol	21.615	C_7_H_8_O	108	74
4	1-Dodecene	27.348	C_12_H_24_	168	83
5	Phenol, 2,4-bis(1,1-dimethylethyl)-	44.440	C_14_H_22_O	206	95
6	Benzophenone	48.725	C_13_H_10_O	182	90
7	(3R,8aS)-3-Methyl-1,2,3,4,6,7,8, 8a-octahydropyrrolo[1,2-a]pyrazine-1,4-dione {Cyclo(L-Pro-D-Ala)}	51.592	C_8_H_12_N_2_O_2_	168	90
8	Pyrrolo[1,2-a]pyrazine-1,4-dione, hexahydro- {Cyclo(Gly-Pro)}	53.131	C_7_H_10_N_2_O_2_	154	96
9	Octahydro-5H,10H-dipyrrolo[1,2-a:1′2’-d]pyrazine-5,10-dione {Cyclo(Pro-Pro)/3,9-diazatricyclo[7.3.0.0(3,7)]dodecan-2,8-dione}	59.248	C_10_H_14_N_2_O_2_	194	64
10	Pyrrolo[1,2-a]pyrazine-1,4-dione, hexahydro-3-(2-methylpropyl)- {Cyclo(Leu-Pro)/1,4-diaza-2,5-dioxo-3-isobutyl bicyclo[4.3.0]nonane}	59.340	C_11_H_18_N_2_O_2_	210	83
11	Pyrrolo[1,2-a]pyrazine-1,4-dione,hexahydro-3-(phenylmethyl)- Cyclo(Phe-Pro)	72.054	C_14_H_16_N_2_O_2_	244	93
12	Phenol, 2,2′-methylenebis[6-(1,1-dimethylethyl)-4-methyl-	73.513	C_23_H_32_O_2_	340	95

## Discussion

According to Goodfellow [59], it is important to acquire data both from DNA-based methods as well as phenotyping in order to provide a sound basis for the taxonomy of the prokaryotes, especially for the genus *Streptomyces*. This is because studies have demonstrated that strains in the genus *Streptomyces* which have high 16SrRNA gene similarity may still display highly varied phenotypic characteristics with regard to their biochemical profiles and carbon source utilization patterns [14, 60]. Therefore, in depth evaluation of biochemical and physiological characteristics of strain MUM265 were conducted to complement the results of phylogenetic analysis, with the aim of providing a better understanding of this strain. The 16S rRNA gene phylogenetic analysis indicated that strain MUM265 does indeed belong to the genus *Streptomyces*. It grows between pH 6.0 and 7.0 (optimum at pH 7), 0 to 6% NaCl concentration (optimum 2%) and temperatures between 20 to 40 °C (optimum at 36 °C). These findings are expected given that these organisms inhabit mangrove environments, which experience daily exposure to tidal inundation with continuously changing salinity and temperature of sediments [61, 62], making a wide range of tolerance to variable conditions of salinity and temperature essential for the survival of this strain. Singh [63] demonstrated that the Biolog Microplate assay not only provides the substrate utilization and growth fingerprint of the test organism but can also be correlated with the production of secondary metabolites with specific bioactivity through additional biological testing and chemical analysis of the extract prepared from each well. In addition, metabolic profile data of strain MUM265 permits the formulation of fermentation media suitable for higher titers and feeding studies [63]. Therefore, this data will be useful in future work on medium optimization which has significant implications on cost in large scale production, as it would facilitate cost saving by enabling the selection of the most cost-effective carbon sources as a substrate. Oxidative stress is a factor that is commonly implicated in the pathogenesis of cancer. Oxidative stress refers to a condition where excessive levels of reactive oxygen species (ROS) are present to such an extent that they overwhelm the body’s antioxidant defense system [64]. ROS are produced by all living organisms as by-products of many normal metabolic functions [65] including the mitochondrial respiratory chain [66], neutrophil-mediated respiratory burst in response to microbial infection, [67] and lipid metabolism in peroxisomes [68]; however, these molecules are highly reactive and can damage cell structures such as carbohydrates, nucleic acids, lipids, and proteins and alter their functions. Therefore, maintaining adequate antioxidant levels within the body to maintain a favourable oxidant/antioxidant balance is an important strategy in the prevention of carcinogenesis; plants have been the focus of recommended antioxidant sources so far [69, 70], but recent work – including this study – support the idea that microorganisms represent an as yet untapped source of potent antioxidants [16, 29, 71].

Oxidation is a very complex process which may occur through a variety of mechanisms [72]; as a result antioxidants may also exert their effects through a variety of pathways. A natural extract could potentially contain a multitude of antioxidants [73] - including phenolic compounds and metal chelators- all of which may have unique mechanisms of action. As a result, it was necessary to utilize a variety of antioxidant assays to gain a better overall picture of the antioxidative power of MUM265 extract; with the assays selected based on the likely mechanisms identified from the existing knowledge/literature. Our results suggest that *Streptomyces* sp. MUM265 extract is a good source of compounds to be developed into antioxidants with dual functions, such as scavenging of free-radicals as well as simultaneously chelating metal ions. This is perhaps unsurprising as these organisms inhabit an extreme environment where they are constantly exposed to abiotic stresses such as high salinity, drought, hypoxia and high ultraviolet radiation [74] and as a result would have needed to develop survival mechanisms in the form of the ability to produce antioxidative metabolites in order to neutralize the environment induced oxidative stress [8]. It is our belief that our findings suggest that *Streptomyces* sp. MUM265 extract has the potential to be used as a chemopreventive agent based on its antioxidant properties.

As an extension to this, we chose to further investigate the cytotoxic effects of MUM265 extract against cancer cells – specifically, against a range of human colon cell lines. We chose to test the extract against a variety of cell lines as each has a unique genetic makeup which may result in variable susceptibility or resistance to the extract. This is likely to be the underlying reason for the differential cytotoxicity observed in the various cell lines during the study following exposure to MUM265 extract. Genetic difference between the cell lines have previously been documented – for example different cell lines possess different variants of the KRAS gene where HT29 and Caco-2 possess wild-type KRAS while SW480 is known to have a mutated KRAS oncogene [75]. Based on this finding, it could be postulated that HT29 and Caco-2 colon cancer cells (with wild-type KRAS) are more sensitive toward MUM265 extract than colon cancer cell SW480 (with mutated KRAS oncogene) which was resistant toward MUM265 extract.

Although ED_50_ was not achieved by the concentrations of MUM265 extract tested in this study, MUM265 extract seems to result in the activation of various pathways that result in apoptosis of vulnerable cells such as Caco-2 cells which showed morphological changes post exposure which were consistent with the activation of apoptosis-related pathways. Additional evidence came in the form of accumulation of sub-G1 cell population which indicated the presence of an increased number of cells with reduced DNA content or fragmented DNA and suggested that the MUM265 extract induced DNA damage in Caco-2 cells, which subsequently led to apoptosis. MUM265 extract also appears to induce loss of MMP in Caco-2 cells; with MMP being a substance which regulates the selectivity and permeability of the mitochondrial membrane towards various substances as well as to maintain mitochondrial functionality [76, 77]. The increased membrane permeability resulting from loss of MMP is also associated with the release of soluble proteins that serve as activators for the caspases and endonucleases responsible for most forms of apoptosis [78]. While all these in combination support our postulation that MUM265 extract’s cytotoxic effect may be mediated via mitochondrial-dependent apoptotic pathways, further mechanistic studies on mitochondrial-dependent cell death pathway would be needed to obtain a clearer understanding of the molecular targets of MUM265 extract.

It is noteworthy that GC-MS analysis has contributed significantly in the bioprospecting of natural products isolated from *Streptomyces* bacteria [28, 79–82]. Most of the compounds detected by GC-MS in MUM265 extract have been also reported previously in *Streptomyces* sp. extracts, such as 2(*5H*)-furanone **(1)** [83], 2-ethyl hexan-1-ol **(2)** [84], benzyl alcohol **(3)** [85], 1-dodecene **(4)** [86], phenol,2,4-bis(1,1-dimethylethyl)- **(5)** [82, 87], benzophenone **(6)** [88, 89], pyrrolo[1,2-a]pyrazine-1,4-dione, hexahydro- **(8)** [16], pyrrolo[1,2-a]pyrazine-1,4-dione, hexahydro-3-(2-methylpropyl)- **(10)** [90], pyrrolo[1,2-a]pyrazine-1,4-dione,hexahydro-3-(phenylmethyl)- **(11)** [90], phenol, 2,2′-methylenebis[6-(1,1-dimethylethyl)-4-methyl- **(12)**.

The detection of phenolic compounds in the *Streptomyces* MUM265 extract is in concordance with the findings of the total phenolic content estimation. The phenolic compounds were phenol,2,4-bis(1,1-dimethylethyl)- **(5)** and phenol, 2,2′-methylenebis[6-(1,1-dimethylethyl)-4-methyl- **(12)**. Given their well-recognized role as antioxidants, phenolic compounds reduce free radicals by means of their hydrogen-donating or electron transferring abilities [91, 92]. Thus, it can be suggested that these phenolic compounds could contribute to the overall antioxidant capacity of *Streptomyces* sp. MUM265 extract, which possess capability to scavenge free-radicals and chelate metal ions. Previously, a study revealed the detection of phenol,2,4-bis(1,1-dimethylethyl)- **(5)** in the crude extract of a *Streptomyces* sp. isolated from vermicompost samples. Interestingly, the crude extract also demonstrated potent antioxidant properties and cytotoxicity against cervical cancer cells [93].

Several cyclic dipeptides have been detected from the extract, including cyclo(L-Pro-D-Ala) or (3R,8aS)-3-methyl-1,2,3,4,6,7,8,8a-octahydropyrrolo[1,2-a]pyrazine-1,4-dione **(7)**, cyclo(Gly-Pro) or pyrrolo[1,2-a]pyrazine-1,4-dione, hexahydro- **(8)**, cyclo(Pro-Pro) or octahydro-5H,10H-dipyrrolo[1,2-a:1′2’-d]pyrazine-5,10-dione **(9)**, cyclo(Leu-Pro) or pyrrolo[1,2-a]pyrazine-1,4-dione, hexahydro-3-(2-methylpropyl)- **(10)** and cyclo(Phe-Pro) or pyrrolo[1,2-a]pyrazine-1,4-dione,hexahydro-3-(phenylmethyl)- **(11)** in the present study. Cyclic dipeptides are known to be a group of the simplest peptide derivatives [94]. Recently, these compounds have also often been reported in studies of fermentation culture of microbes [18, 95, 96]. Extracts of *Streptomyces* sp. which contain these cyclic dipeptides have previously been demonstrated to exhibit promising antioxidant activity [16, 29]. Besides the detection of cyclic dipeptides in *Streptomyces*, pyrrolo[1,2-a]pyrazine-1,4-dione, hexahydro- **(8)** was also reported from sponge associated *Bacillus* sp. and it was shown to reduce oxidative damage by radicals [97]. Furthermore, the extract of *Micrococcus lutues* containing both pyrrolo[1,2-a]pyrazine-1,4-dione, hexahydro- **(8)** and pyrrolo[1,2-a]pyrazine-1,4-dione, hexahydro-3-(2-methylpropyl)- **(10)** was demonstrated to exert cytotoxic effect on HCT15 [98]. Additionally, cyclo(L-Pro-L-Phe) **(11),** one of the cyclic dipeptides present in a mixture isolated from *Pseudomonas aeruginosa* PAO1 strain was shown to promote cell death in HeLa and Caco-2 cells in a dose-dependent manner. The study also suggested that the mixture - which contained cyclic dipeptides - mediated the apoptotic pathway related inhibition of cell proliferation [96]. Similarly, cyclo(L-Pro-L-Phe) **(11)** isolated from *Bacillus* species also caused apoptosis in U-87MG cells from human glioblastoma through AKT1 inactivation by down-regulating the phosphorylation of AKT1 serine/theorine kinase at 0.01 mg/mL [95, 99]. These findings correlate well with the cytotoxic effect of MUM265 extract against Caco-2 cells, suggesting that the cyclic dipeptides present in the extract could have induced apoptosis in Caco-2 cells. Perhaps purification of these compounds could be performed in future to evaluate their efficacy by determining the ED50 against colon cancer cells.

The GC-MS chemical profiling of MUM265 extract detected chemical constituents with well recognized antioxidant and cytotoxic properties. These findings suggest that these chemical constituents could have contributed to both antioxidant capacity and cytotoxic properties of *Streptomyces* sp. MUM265 extract. On the whole, the detection of these groups of compounds in MUM265 extract further strengthens the prospect of mangrove derived *Streptomyces* being recognized as a producer of compounds that may be potential drug candidates for chemoprevention of cancer.

## Conclusions

The extensive characterization of *Streptomyces* sp. MUM265 conducted in this study indicates it is a potential source of novel industrial and pharmaceutical products. MUM265 has several features that make it highly attractive from an industry view point, chiefly its good salt and temperature tolerance, as well as its secretion of industrially valuable enzymes and production of bioactive compounds. We believe that our findings regarding the behavior and substrate utilization of the strain and its bioactivities, are potentially key to further manipulation to exploit the biosynthetic output of the strain as they will aid with creation of conditions stimulating maximum efficiency in producing the desired compounds. The antioxidant and anti-colon cancer properties demonstrated in our work make this organism well worth studying further. The MUM265 extract exhibits antioxidant properties including DPPH radical scavenging, ABTS radical scavenging, superoxide anion radical scavenging and metal chelating activity. In addition, MUM265 extract induces depolarization of mitochondrial membrane potential and DNA fragmentation of Caco-2 cells, suggesting apoptosis-inducing properties of MUM265 extract in colon cancer cells. This raises the exciting possibility that further exploration of the biochemical properties and mechanisms of action of the various compounds in the extract from this organism will reveal new mechanisms of action for chemopreventive and anti-cancer drug development.

## Methods

### Isolation of strain MUM265 from mangrove soil

The isolation of strain MUM265 was conducted on a mangrove soil sample of Kuala Selangor mangrove forest, Malaysia (3° 21′ 45.8″ N 101° 18′ 4.5″ E) in Jan 2015. The soil samples were collected from the soil layer within the top 20 cm depth with the removal of surface to a 2 to 3 cm depth, stored in sterile plastic bags and kept at − 20 °C. The soil samples were air-dried prior to grinding with mortar and pestle. Wet heat pretreatment of the soil samples was performed for 15 mins at 50 °C as described by Takahashi et al. [100]. Ten times dilution of the pre-treated soil samples were spread onto the ISP 2 isolation medium to which cycloheximide (25 μg/ml) and nystatin (10 μg/ml) had been added, and incubated at 28 °C for 14 days [101]. Pure culture of strain MUM265 was isolated and maintained on slants of ISP 2 agar at 28 °C and in 20% (*v*/v) glycerol stock at − 20 °C.

### Genomic DNA extraction, 16S rRNA PCR and phylogenetic analysis

Genomic DNA extraction was performed on strain MUM265 prior to 16S rRNA sequencing [8]. The 16S rRNA gene was amplified by PCR according to the method of Lee et al. [14] using the primer pair 27F-1492R [9]. The PCR reactions were conducted based on the protocol for SolGent™ 2X Taq PLUS PCR Smart mix using the Kyratex PCR Supercycler (Kyratec, Australia). The optimized cycling conditions were as follows: (i) 95 °C for 5 min, (ii) 35 cycles of 94 °C for 50 s, 55 °C for 1 min and 72 °C for 1 min 30 s; and (iii) 72 °C for 8 min. CLUSTAL-X software was used to align the 16S rRNA gene sequence of strain MUM265 with the 16S rRNA sequences of related *Streptomyces* type strains retrieved from the GenBank/EMBL/DDBJ databases [102]. Phylogenetic trees were built via neighbour-joining algorithm [103] (Fig. 1) using MEGA version 6.0 [104] based on the Kimura’s two-parameter model [105]. The EzTaxon-e server (https://www.ezbiocloud.net/) [106] was used to determine sequence similarity. A 1000 bootstrap resampling based on method of Felsenstein [107] was performed to assess the clade support of the constructed trees topologies.

### Phenotypic characteristics

The colony morphology and phenotypic characteristics of strain MUM265 were examined using different agar media (HiMedia, India) including ISP 2, ISP 3, ISP 4, ISP 5, ISP 6, ISP 7 [101], actinomycetes isolation agar (AIA) [108], starch casein agar (SCA) [109] and nutrient agar [110] for 14 days at 28 °C. The colony color of strain MUM265 was determined with reference to ISCC-NBS color charts [111]. Microscopic evaluation was performed with a light microscope (80i, Nikon) and scanning electron microscope (TM-1000, Hitachi) on 7–14 days culture. Gram staining was conducted [112]. The production of melanoid pigments on ISP7 media was determined as described by Lee et al. [113]. The effect of temperature on the growth of strain MUM265 was assessed on ISP2 agar. The effects of salt concentration and pH on the growth of strain MUM265 were assessed in tryptic soy broth [29]. Hemolytic activity of strain MUM265 was examined on horse blood agar [114]. Enzymatic tests were performed to test the enzymes produced by strain MUM265 [115]. Antibiotic susceptibility of strain MUM265 was examined using the antibiotic disc diffusion method [116]. Biolog GenIII MicroPlates was used to evaluate the carbon and nitrogen source utilization and chemical sensitivity of the strain (Biolog, USA).

### Extract preparation of strain MUM265

Fermentation of strain MUM265 was performed according to Tan et al. [117] using Han’s Fermentation Media 1 (HFM1) (Biomerge, Malaysia). Extraction was conducted on the freeze-dried fermentation product according to Tan et al. [117]. The extract was collected and suspended in dimethyl sulphoxide (DMSO) before further analysis.

### Antioxidant activity evaluation of MUM265 extract

DPPH (2,2-diphenyl-1-picrylhydrazyl) radical scavenging assay was conducted according to the previously described method [16]. The reduced absorbance of DPPH radical was measured at 515 nm with a microplate reader. The superoxide anion scavenging activity or superoxide dismutase (SOD) like activity of MUM265 extract was examined by employing 19,160 SOD Assay Kit-WST (Sigma Aldrich) with slight modification [29]. The 2,2′-azino-bis(3-ethylbenzothiazoline-6-sulphonic acid) (ABTS) assay was performed as described in Tan et al. [29]. ABTS radical cation (ABTS^●^+) was generated by reacting 7 mM ABTS stock solution with 2.45 mM potassium persulphate for 24 h prior to conducting the assay. The reduction of ABTS radical absorbance was measured at 734 nm with a microplate reader. Metal-chelating activity was measured as described by Tan et al. [118]. The TPC in MUM265 extract was assessed by using Folin-Ciocalteu’s reagent method [119]. Meanwhile, the total flavonoid content in MUM265 extract was assessed by the aluminium chloride colorimetric 96 well-microplate method [120].

### Cell lines maintenance and growth condition

Caco-2, HT-29, SW480 and CCD-18Co cell lines were obtained from the American Type Culture Collection (ATCC, Manassas, VA, USA). All cells were cultured in RPMI-1640 (Gibco) supplemented with 10% fetal bovine serum and 1x antibiotic-antimycotic (Gibco) at 37 °C in a CO_2_ incubator.

### Cell treatment and MTT cell viability assay

The cytotoxic effects of *Streptomyces* sp. MUM265 extract on human colon cancer cell lines were evaluated using 3-(4,5-dimethylthiazol-2-yl)-2,5-diphenyltetrazolium bromide (MTT) assay based on a previously established method [121]. Cells were seeded into 96-well plate at density of 5 × 10^3^ cells per well and left in incubator overnight. Control cells were treated with 0.5% DMSO while the concentration of DMSO was maintained at 0.5% across all treatments with MUM265 extract for the entire series of concentrations ranging from 25 to 400 μg/mL. MTT assay was conducted after 72 h of exposure to the extract. The assay was performed by adding 20 μL of MTT solution (5 mg/mL) to each well and incubated at 37 °C with 5% CO_2_ for 4 h. The medium was removed prior to the dissolution of the formazan product formed using 100 μL of DMSO. The amount of MTT-formazan was determined at 570 nm absorbance with 650 nm as reference wavelength.

### Cell cycle analysis

DNA content and cell cycle distribution were evaluated using propidium iodide (PI) staining [121]. After exposure to MUM265 extract for 24 h, cells were harvested and washed with PBS before being fixed with 70% ice-cold ethanol at -20 °C overnight. Fixed cells were washed twice and stained in a buffer containing 25 μg/mL PI, 0.1% Triton-X-100 and 100 μg/mL RNase A for 30 mins in the dark at room temperature. The PI-stained cells were analyzed using BD FACSVerse™ flow cytometer (BD Bioscience, San Jose, CA).

### Mitochondrial membrane potential

JC-1 (5,5′,6,6′-tetrachloro-1,1′,3,3′-tetrathylbenzimidazolcarbocyanine iodide) dye from BD™ MitoScreen kit ((BD Bioscience, San Jose, CA) was used to evaluate the status of mitochondrial membrane potential of the cells. After exposure to MUM265 extract for 24 h, cells were harvested and washed twice before analysis using BD FACSVerse™ flow cytometer (BD Bioscience, San Jose, CA).

### Gas chromatography-mass spectrometry (GC-MS) analysis

GC-MS chemical profiling was conducted based on a previously developed protocol [122]. The analysis was conducted using Agilent Technologies 6980 N (GC) equipped with 5979 Mass Selective Detector (MS), HP-5MS (5% phenyl methyl siloxane) capillary column of dimensions 30.0 m × 250 μm × 0.25 μm using helium as the carrier gas at 1 mL/ min. The experiment was initiated by maintaining the column temperature at 40 °C for 10 mins, followed by an increase of 3 °C per min to 250 °C and was kept isothermal for 5 min. The MS was operating at 70 eV. Comparison of the mass spectral between the detected chemical constituents and the standards available in W9 N11 MS library was performed to identify the chemical constituents present in the extract.

### Statistical analysis

Both antioxidant and cytotoxicity assays were conducted at least in triplicate. Results were expressed in mean ± standard deviation (SD). The statistical analysis was performed using SPSS software. The difference between the treated and untreated groups was determined by one-way analysis of variance (ANOVA) and Tukey’s post hoc analysis. A statistically significant difference was determined when *p* ≤ 0.05. Pearson’s correlation analysis was used to evaluate the relationship between the phenolic content and the antioxidant activity of the extract [123].

## Additional file

Additional file 1: **Table S1.** Antibiotic susceptibility test. **Figure S1.** The mass spectrum of the constituents **(1-12)** identified from the GC-MS analysis. (a) The mass spectrum of the constituents obtained from MUM265 extract, (b) the mass spectrum of the standard compounds available on W9N11 MS library. (DOCX 559 kb)

## Abbreviations

ABTS: 2,2′-azino-bis(3-ethylbenzothiazoline-6-sulphonic acid
DPPH: 2,2-diphenyl-1-picrylhydrazyl
GC-MS: Gas chromatography mass spectrometry
ISP: International Streptomyces Project
SOD: Superoxide oxide dismutase
TPC: Total phenolic content
