# Supplementary material for: Mangrove derived Streptomyces sp. MUM265 as a potential source of antioxidant and anticolon-cancer agents
Source: BMC Microbiol. 2019 Feb 13;19:38. doi: 10.1186/s12866-019-1409-7 (PMC6375222; doi:10.1186/s12866-019-1409-7)
Supplement: Supplementary file 1 — Table S1. Antibiotic susceptibility test. Figure S1. The mass spectrum of the constituents (1-12) identified from the GC-MS analysis. (a) The mass spectrum of the constituents obtained from MUM265 extract, (b) the mass spectrum of the standard compounds available on W9N11 MS library. (DOCX 559 kb) [file 12866_2019_1409_MOESM1_ESM.docx]

Table S1. Antibiotic susceptibility test.

| Antibiotic discs | Zone of inhibition |
| --- | --- |
| Gentamicin (120ug) | 33mm |
| Chloramphenicol (30ug) | 16mm |
| Erythromycin (15ug) | 32mm |
| Vancomycin | 29mm |
| Cefotaxime (30ug) | 10mm |
| Penicillin G | No zone |
| Tetracycline (30ug) | 10mm |
| Ampicillin (10ug) | No zone |
| Ampicillin /salbactam | 13mm |
| Nalidixic acid (30ug) | No zone |

Figure S1. The mass spectrum of the constituents **(1-12)** identified from the GC-MS analysis. (a) The mass spectrum of the constituents obtained from MUM265 extract, (b) the mass spectrum of the standard compounds available on W9N11 MS library.


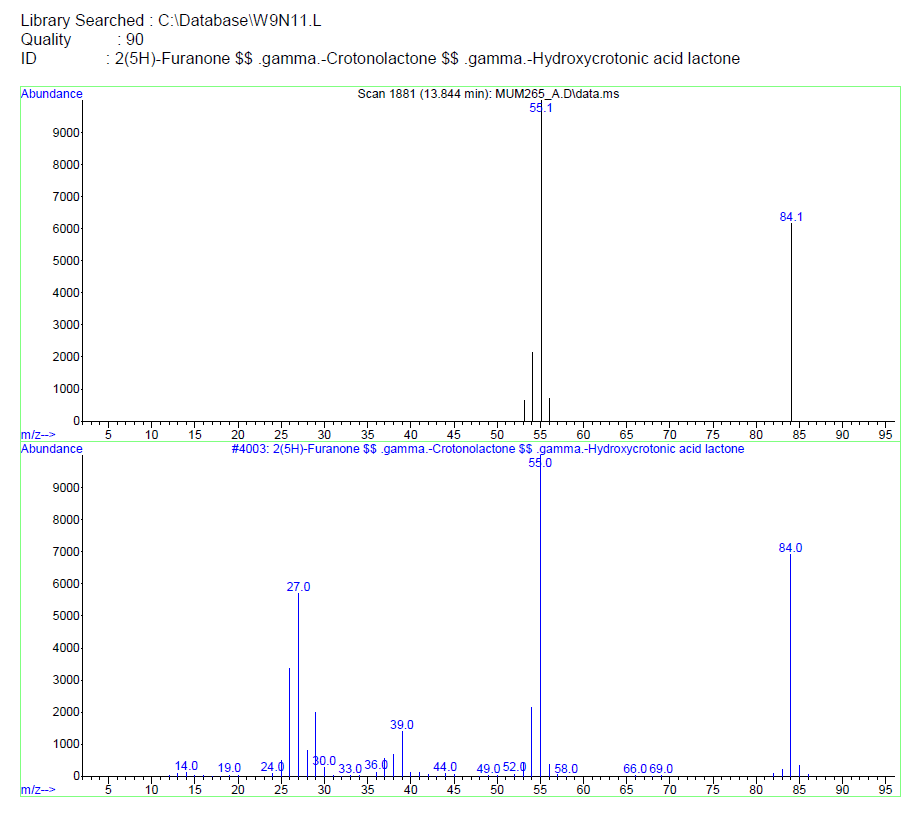


**1 (b)**

**1 (a)**


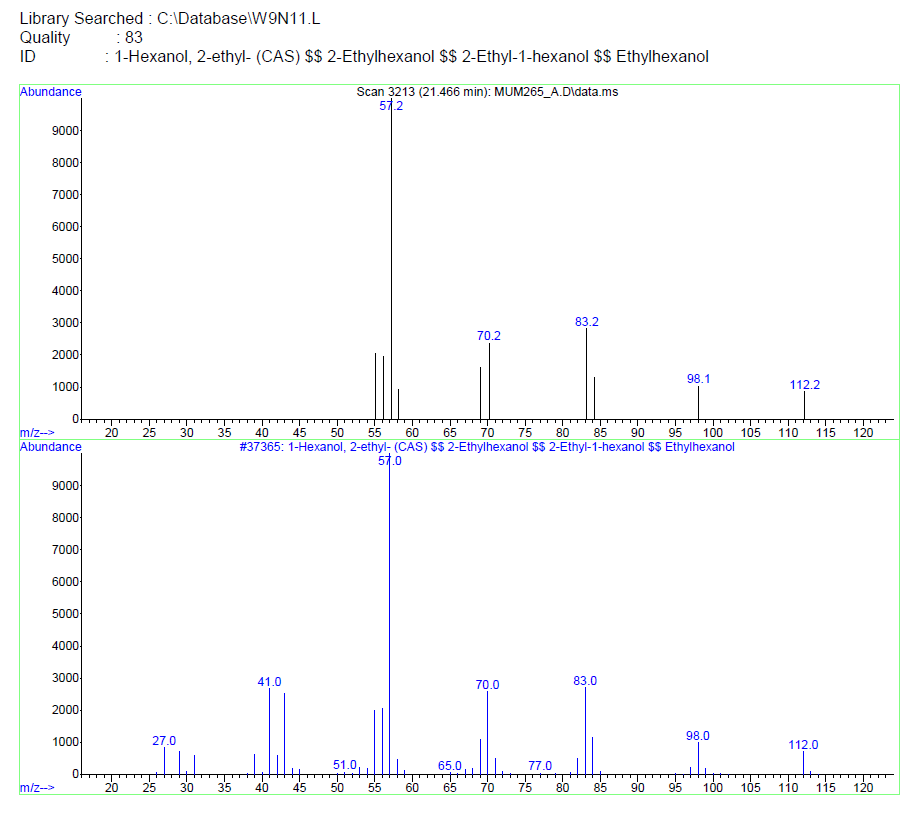


**2 (b)**

**2 (a)**


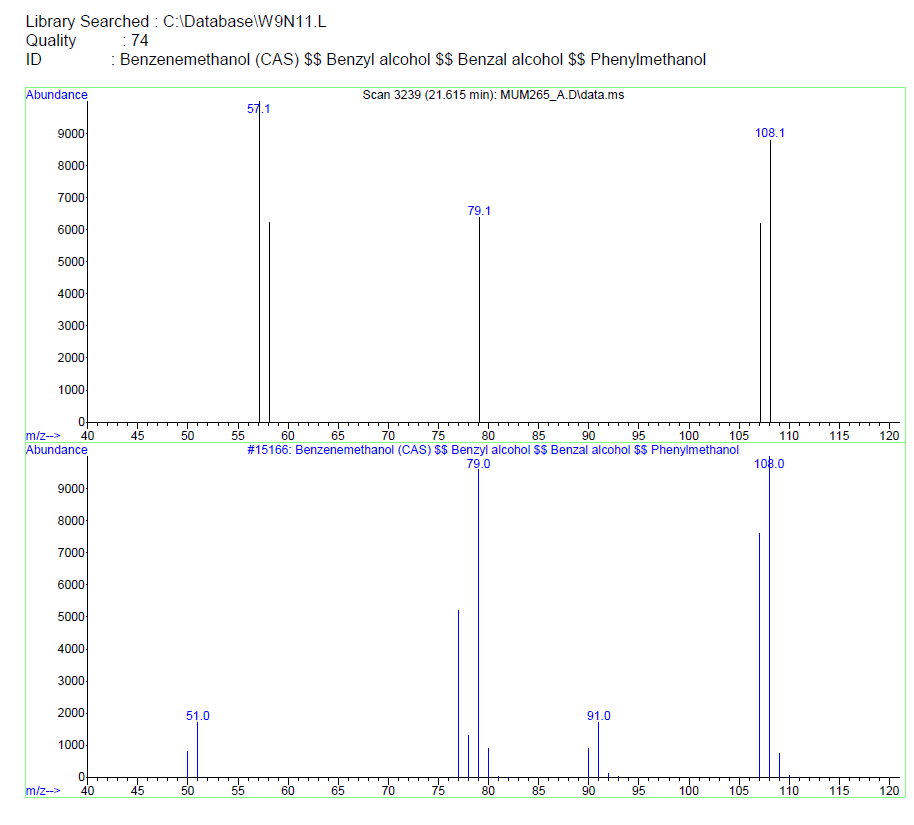


**3 (a)**

**3 (b)**


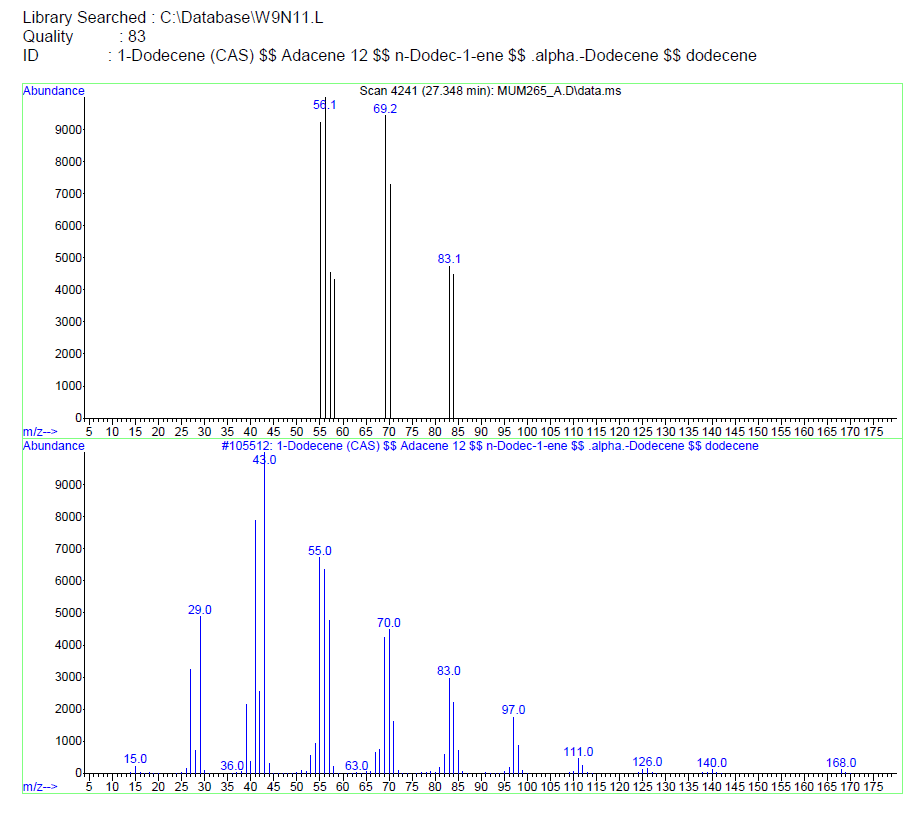


**4 (a)**

**4 (b)**


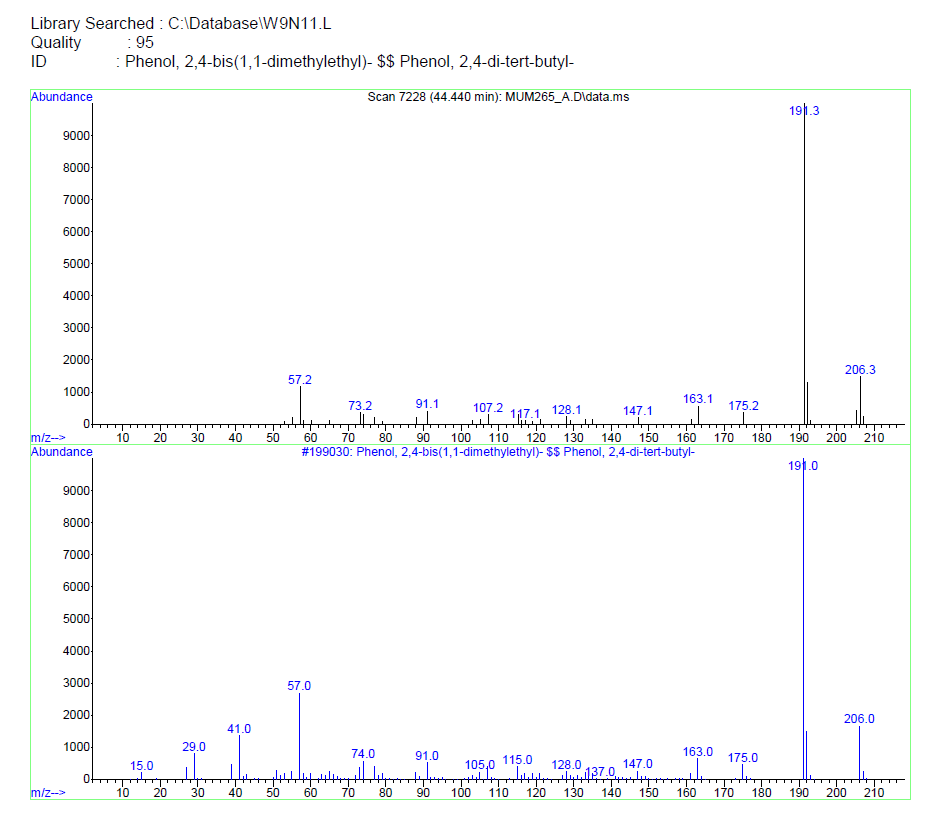


**5 (a)**

**5 (b)**


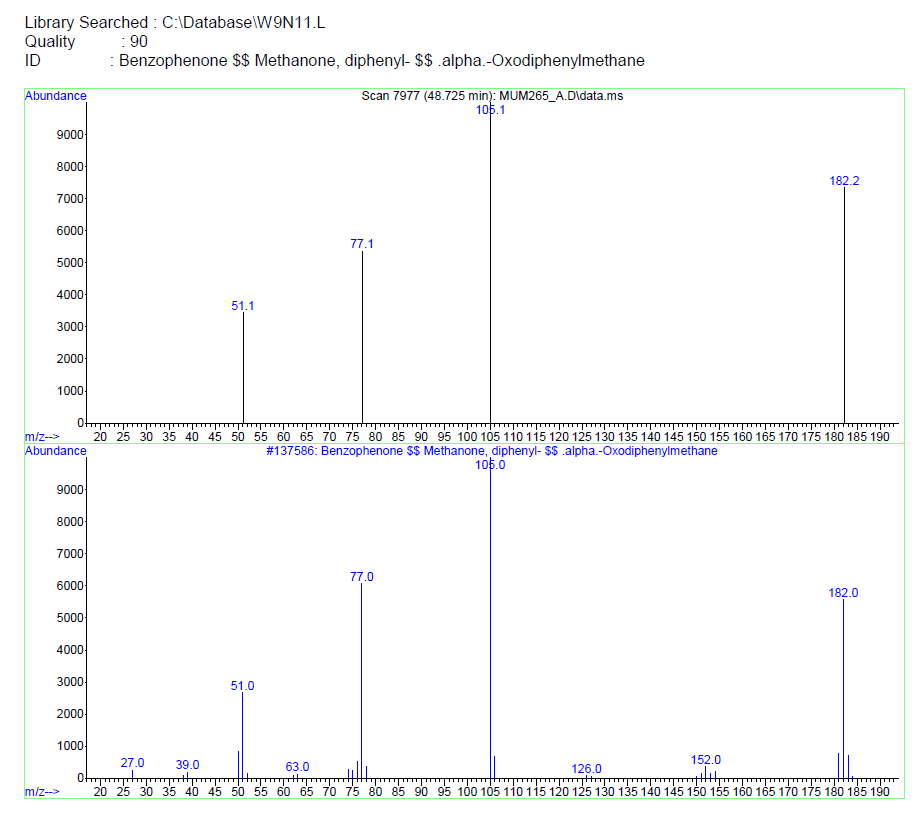


**6 (a)**

**6 (b)**


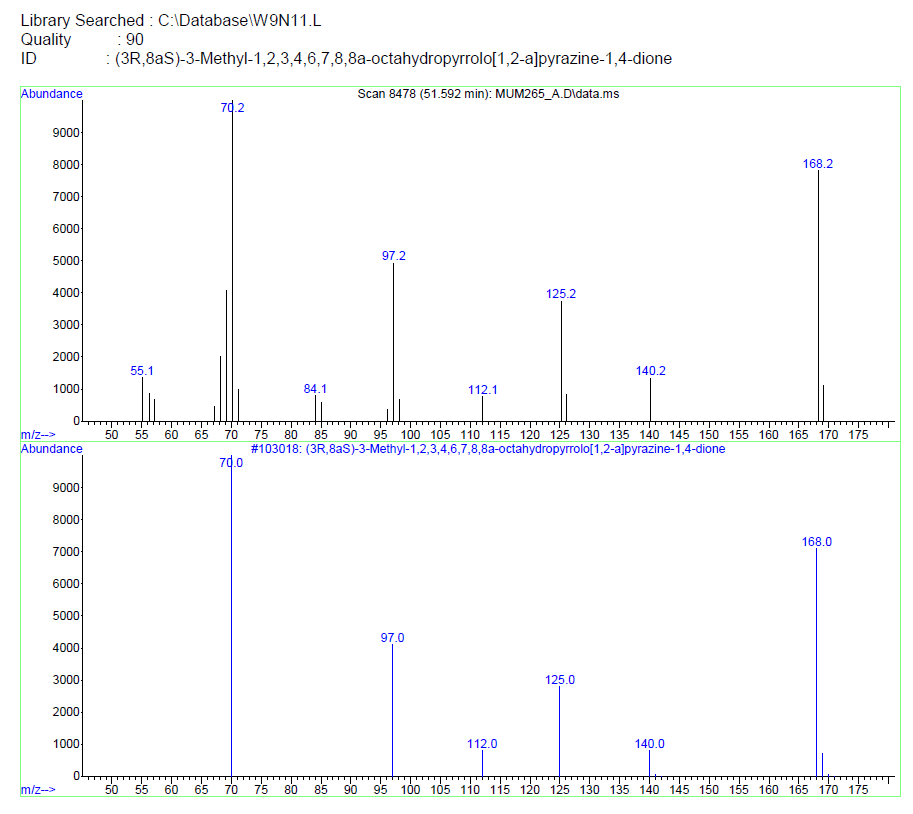


**7 (a)**

**7 (b)**


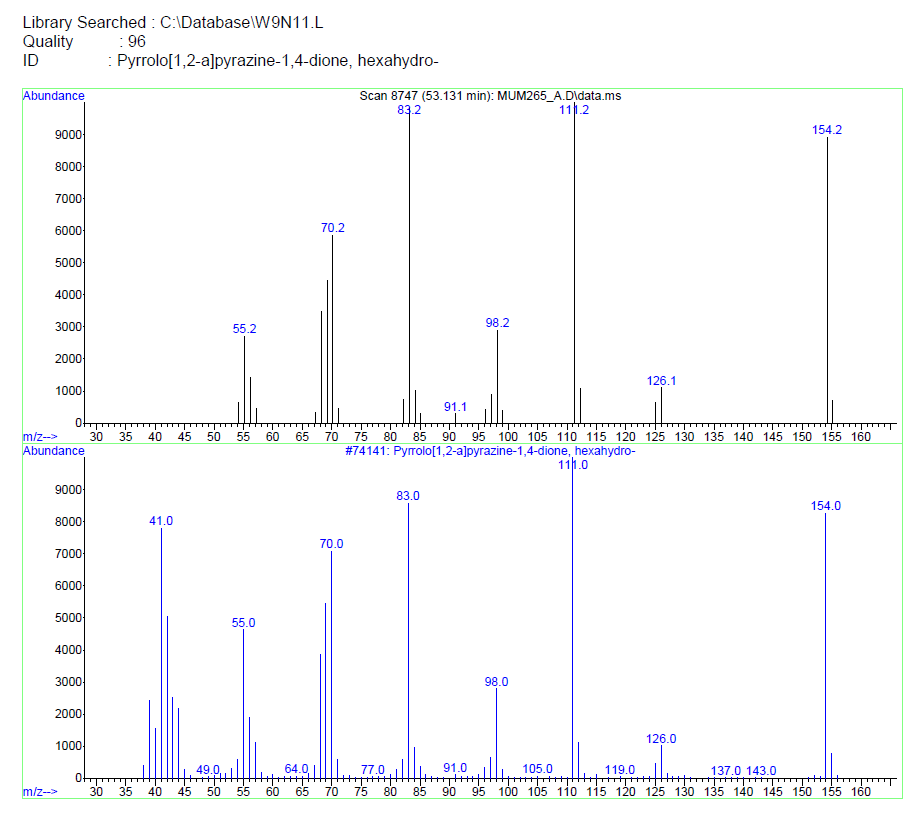


**8 (a)**

**8 (b)**


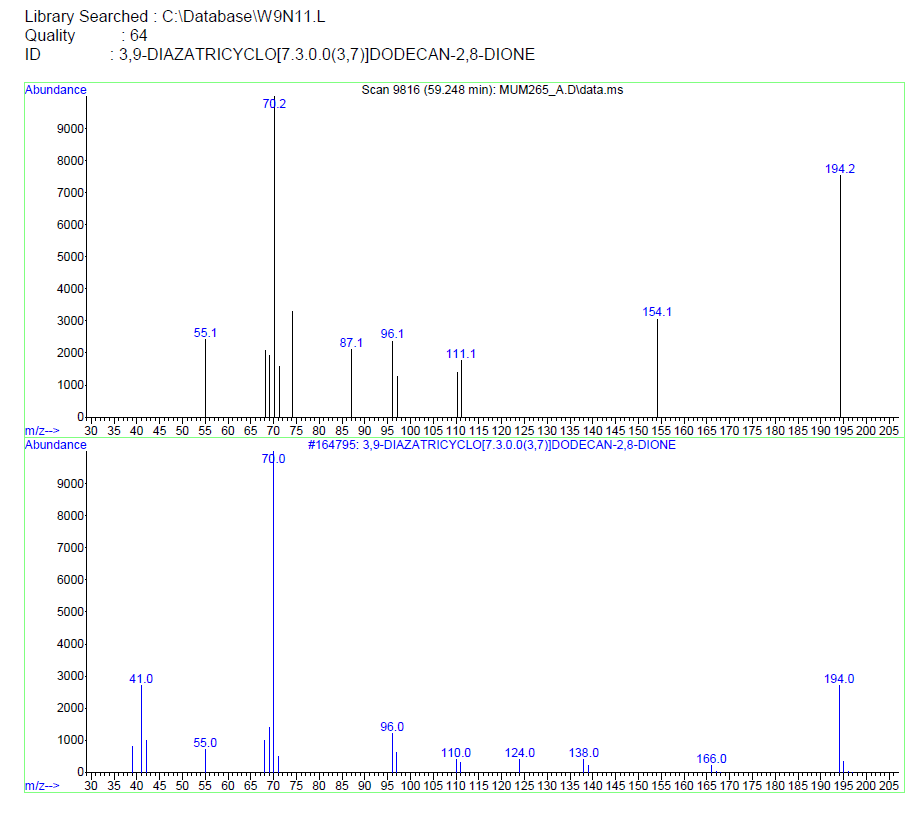


**9 (a)**

**9 (b)**


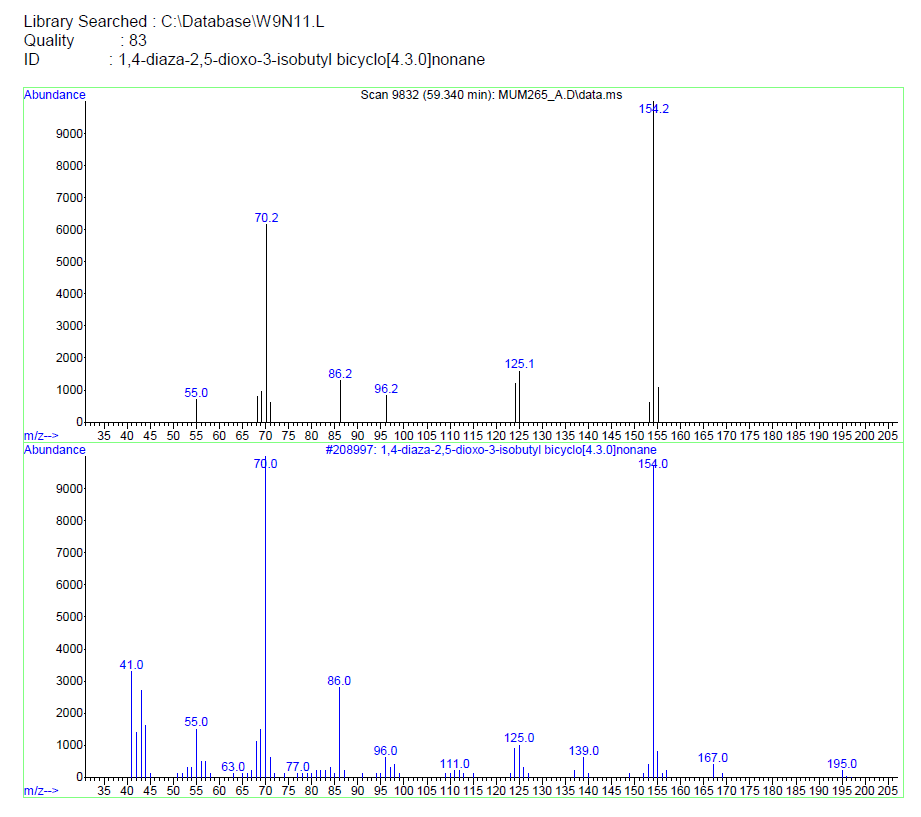


**10 (a)**

**10 (b)**


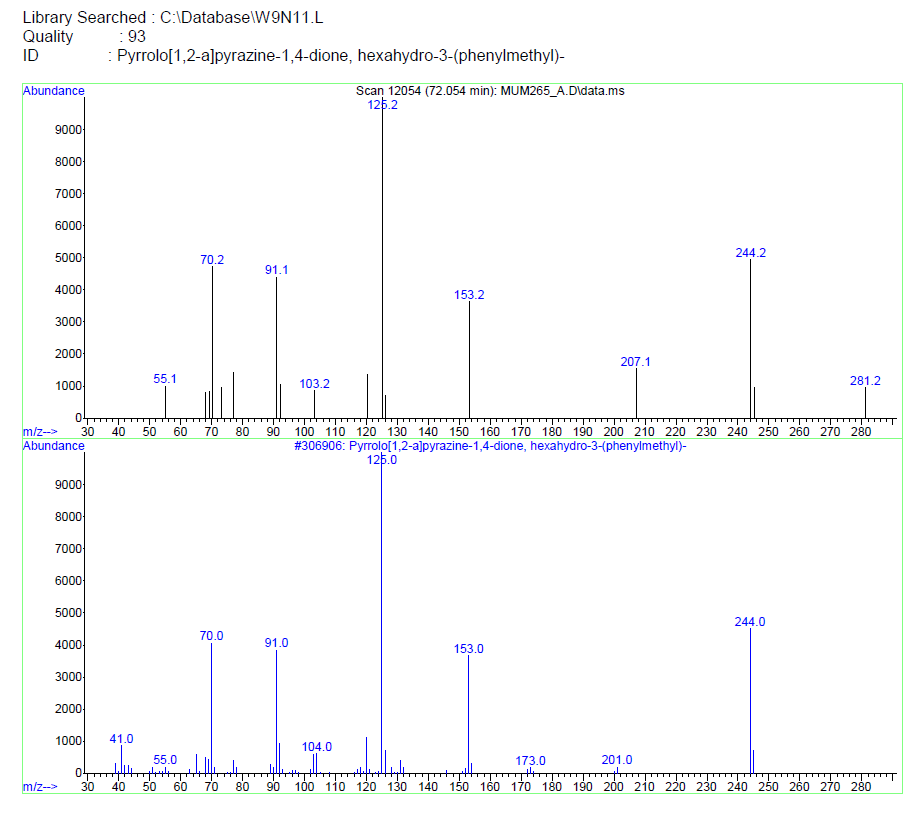


**11 (a)**

**11 (b)**


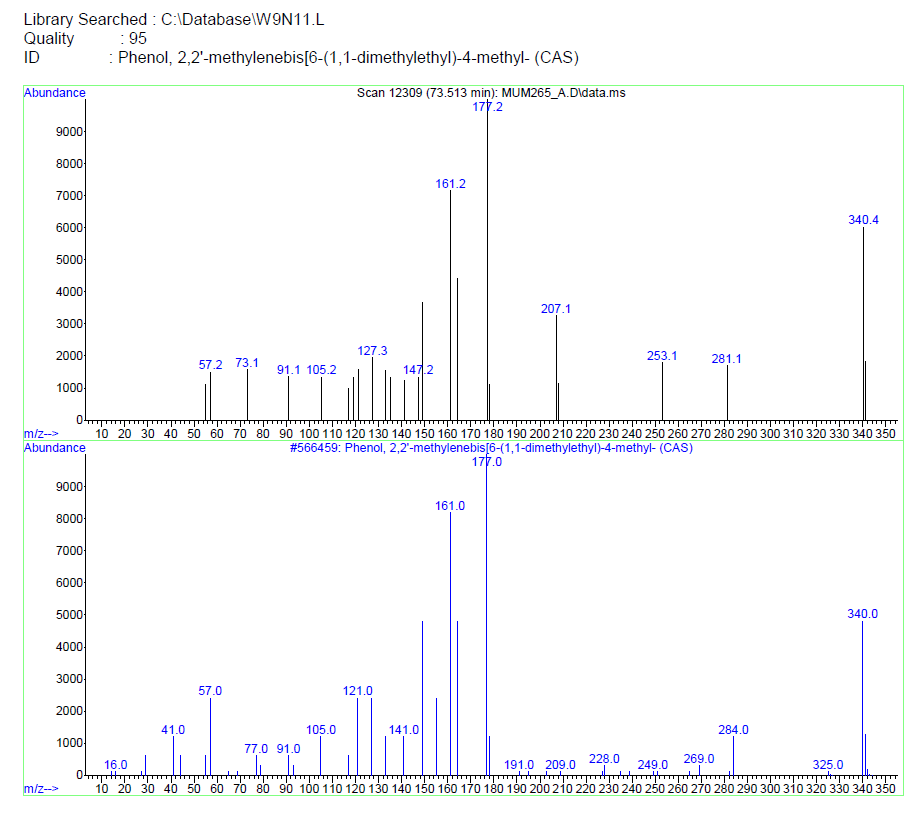


**12 (a)**

**12 (b)**
